# Reinforcement Learning-Based Nonlinear Model Predictive Controller for a Jacketed Reactor: A Machine Learning Concept Validation Using Jetson Orin

**DOI:** 10.1021/acsomega.5c03219

**Published:** 2025-07-09

**Authors:** Aishwarya Selvamurugan, Aromal Vinod Kumar, Hrishikesh R. Palan, Arockiaraj Simiyon, Thirunavukkarasu Indiran

**Affiliations:** † Department of Computer Science Engineering, 306155 Sri Eshwar College of Engineering, Coimbatore 641202, Tamil Nadu, India; ‡ Machine Learning for Advanced Process Control Lab, Department of Instrumentation and Control Engineering, 76793 Manipal Institute of Technology, Manipal Academy of Higher Education, Manipal 576 104, Karnataka, India; § Manipal School of Information Science, Manipal Academy of Higher Education, Manipal 576 104, Karnataka, India

## Abstract

In this research work authors have experimentally validated a blend of Machine Learning and Nonlinear Model Predictive Control (NMPC) framework designed to track the temperature profile in a Batch Reactor (BR) with an actor-critic reinforcement learning (A2CRL) methodology for dynamic weight updates. Recurrent Neural Network (RNN)-based approach for modeling is used for the open loop data collected from the lab scale batch reactor. Batch reactors are extensively utilized in industries like specialty chemicals, pharmaceuticals, and food processing because of their adaptability, especially for small-to-medium-scale production, intricate reaction dynamics, and diverse operational conditions. Thermal runaway in batch reactor is still an open-ended problem in process industry to address. The actor-critic method proficiently integrates policy optimization and value function estimates to dynamically regulate the heat produced by exothermic reactions. RNNs are employed to capture temporal dependencies in the system dynamics, enabling more accurate predictions and efficient control actions. The proposed framework is trained using open-loop experimental data and optimized to dynamically adjust the coolant flow rate, ensuring precise temperature regulation and stability. Compared to existing deep learning-based NMPC implementations, the proposed actor-critic methodology enhances NMPC controller performance by balancing prediction accuracy and real-time computational efficiency. Results demonstrate significant improvements in process efficiency, energy consumption reduction, and operational safety, validating the potential of this approach for deployment in industrial-scale batch reactor systems.

## Graphical abstract

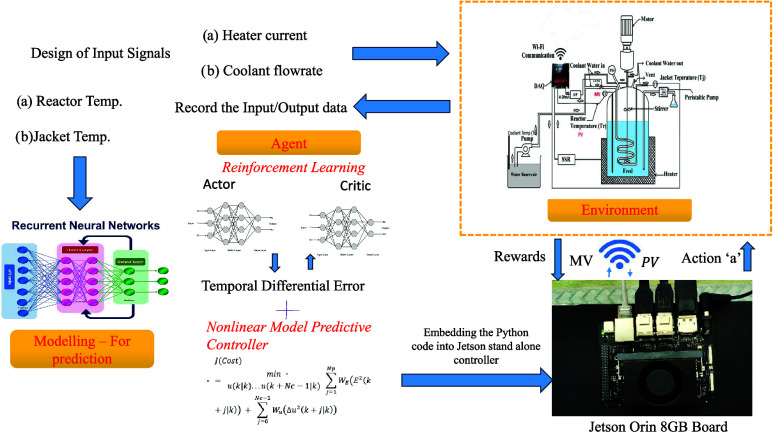

## 1 Introduction

Batch reactors (BRs) are extensively utilized in chemical and pharmaceutical industries for their adaptability in processing diverse chemical reactions and producing high-value products.^1^ However, the nonlinear dynamics, time-dependent operational constraints, and sensitivity to external disturbances in BRs make precise control challenging.

Control strategies have included machine learning (ML) approaches to enhance responsiveness and efficiency. This action was spurred by the increased demand for sophisticated control measures. Despite the increasing use of ML in real-time systems, there are still problems to be addressed in terms of stability and learning efficiency. The industry is seeking solutions that possess the ability to adapt to changing conditions while simultaneously retaining optimal performance. Traditional control systems typically rely mostly on static features and do not incorporate real-time feedback, resulting in reduced efficiency and an elevated error rate. These deficiencies were especially worrisome in relation to the difficulties of overseeing complicated systems.

BRs are widely used in the process industry for a variety of chemical processes. The BR was operated using the coolant flow rate “Fc” and heater current “*H*” as inputs. The coolant flow rate varies from 0.25 to 0.75 mL/min, while the *H* goes from 4 to 20 mA. The temperature of the reactor (*T*_r_), the jacket (*T_j_*), and the coolant (*T_c_*) varied within the range of 0 to 100 °C as the output of the BR system. Figure 1 depicts the Pilot plant BR with Jetson Orin for validating ML algorithm in the “Machine Learning for Advanced Process Control Lab, MIT, Manipal”. This work employs the experimental setup and system dynamics utilized in ref 1 by the same authors. All the system dynamics are explained in this article. Controlling the BR’s temperature to achieve appropriate operating conditions is critical for ensuring the operation’s effectiveness and safety.^2–4^

**Figure 1:**
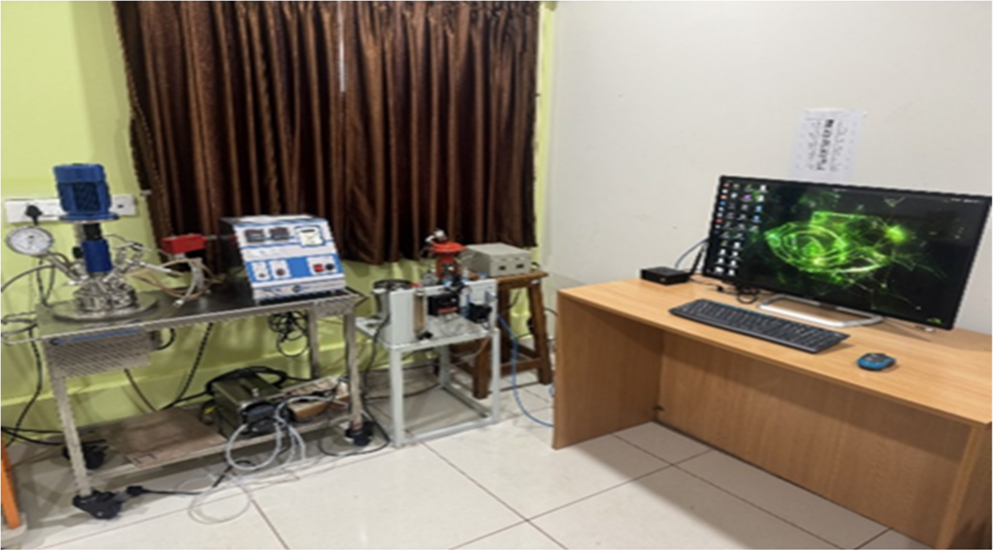
Pilot plant BR with Jetson Orin for validating ML algorithm in “Machine Learning for Advanced Process Control Lab, MIT, Manipal”. Image captured by Thirunavukkarasu Indiran.

Traditional control approaches, like Proportional-Integral-Derivative (PID) controllers, though widely adopted, lack adaptability to changing system conditions and fail to handle nonlinearities effectively. Similarly, traditional Model Predictive Control (MPC) methods, while more capable of managing constraints and optimizing performance, are computationally demanding and heavily dependent on accurate system models, which limits their application to highly dynamic or uncertain systems.^5,6^

Reinforcement learning (RL) tactics have gained recognition in recent years as a highly successful technique for efficiently addressing these challenges. This article proposes a novel approach by integrating RL with Nonlinear Model Predictive Control (NMPC), leveraging RL’s ability to adapt to changing system dynamics and uncertainties. By combining RL’s data-driven adaptability with NMPC’s optimization-based predictive control, this method overcomes the shortcomings of traditional controllers, enhancing robustness and efficiency in nonlinear process control scenarios.^7,8^

The article is organized in the following manner: Section 2 provides an in-depth analysis of the theoretical basis for the RL technique utilized in this work. Section 3 provides a comprehensive explanation of the methods utilized in this study: the formulation of the RNN model, the actor-critic (A2C) framework, the algorithm for NMPC parameter optimization, and the integration of RNN with MPC. Section 4 delineates real-time validation. Section 5 presents a conclusion and outlines prospective research directions.

## 2 Background Theory

This section discusses the background theory and literature survey used in this article. In the field of ML, RL is a powerful methodology. This is particularly true for a group of algorithms that help with making decisions over time in complex real-time systems with nonlinear dynamics.

### 2.1 Reinforcement Learning

Reinforcement learning (RL) operates in a dynamic environment to determine the best sequence of actions to achieve the optimal goal. This is accomplished by enabling a software component known as an agent to explore, interact with, and learn from the environment, as shown in Figure 2. The learning occurs through interaction with the environment, leading to the achievement of optimal goals related to the environment’s state. A reward signal determines the goal, which requires maximization. The agent must have the ability to partially or fully sense the environment’s state and take actions to affect the environment’s state through interaction. An agent learns to behave in an environment by performing actions and receiving feedback on its actions in the form of rewards. This article employs the RL algorithm that uses separate NNs to estimate the policy by the actor network (ATN) and the value function by the critic network (CRN).^4,9^

**Figure 2:**
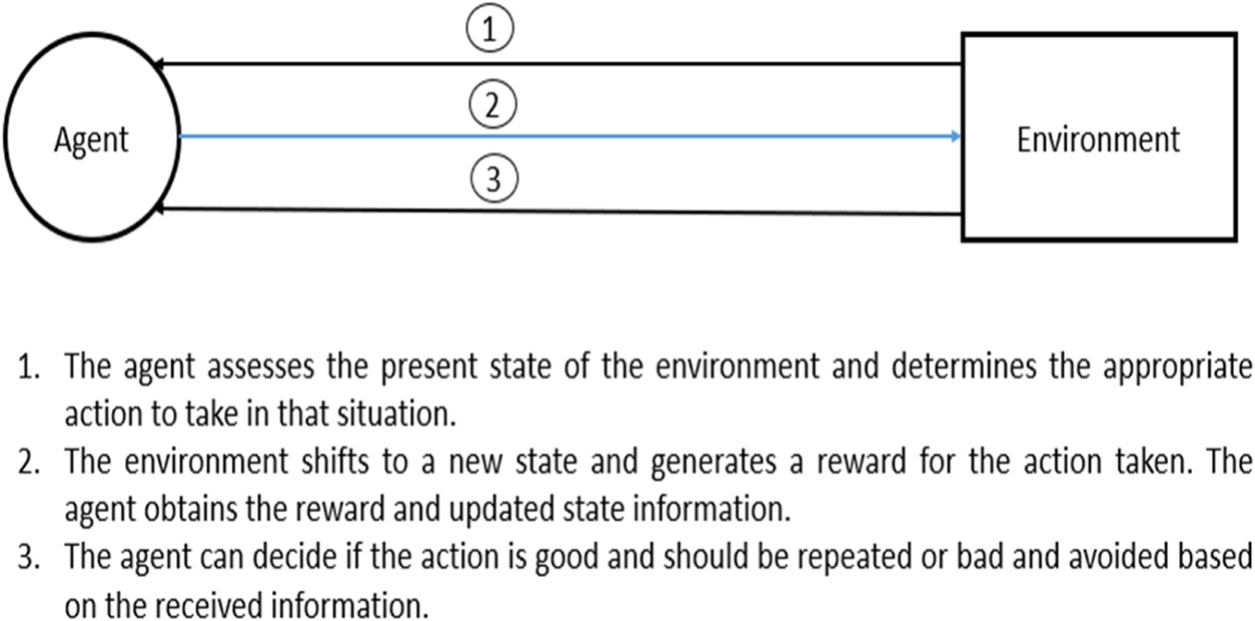
Agent and environment interaction.

#### 2.1.1 Elements of Reinforcement Learning

The five essential aspects to consider in reinforcement learning are environment, reward, policy, training, and deployment. This section offers a concise elucidation of these words.^10^

##### 2.1.1.1 Environment

This includes all entities and phenomena that exist outside of the agent. From the BR controls’ point of view, the environment includes the system’s dynamics. This is the location where the agent transmits actions while concurrently producing rewards and observations.

##### 2.1.1.2 Reward Signal

After creating the environment, the next step involves determining the desired actions and the corresponding rewards for the agent. To get the desired outcome, it is necessary to create a reward function that enables the learning algorithm to recognize improvements in the policy and eventually reach the desired result through convergence. A reward can be designed considering actions, states, and errors between the desired state and the actual state. A reward signal defines the goal in a RL problem. The reward signal thus defines what the correct and incorrect events are for the agent. Equation 1 shows a sample reward design as follows^11^

1 $$r_{t}=\{\begin{array}{l} +10,\mathrm{if}\;\left(s^{*}-s\right)\leq 3\\ +1,\mathrm{elif}\;\left(s^{*}-s\right)\leq 5\\ -10|s^{*}-s|,\mathrm{else} \end{array}$$

##### 2.1.1.3 Policy

A policy establishes the learning agent’s behavior at a specific moment, effectively mapping observed environmental states to appropriate actions. Two components form the agent: the policy and the learning algorithm. The policy functions as a mathematical function, assigning actions to observations, whereas the learning algorithm employs a computing technique to determine the most optimal policy. Both concepts are interrelated.^11^

##### 2.1.1.4 A Universal Function Approximator

The nodes in the artificial neural network have the ability to approximate universal functions. Therefore, by selecting the appropriate number of nodes and layers, it is possible to construct the network to replicate any specified input and output relationship. Although the function may be a bit complex, the universal nature of neural networks ensures that they can carry it out.^12^

##### 2.1.1.5 Policy Network

In Figure 3, the actor network architecture consists of 1 input, 1 output, and 2 hidden layers, with each hidden layer containing 3 nodes. In a fully connected network, every input node uses weighted connections to connect to each node in the subsequent layer. This pattern continues from one layer to the next until reaching the output nodes.^11^

**Figure 3:**
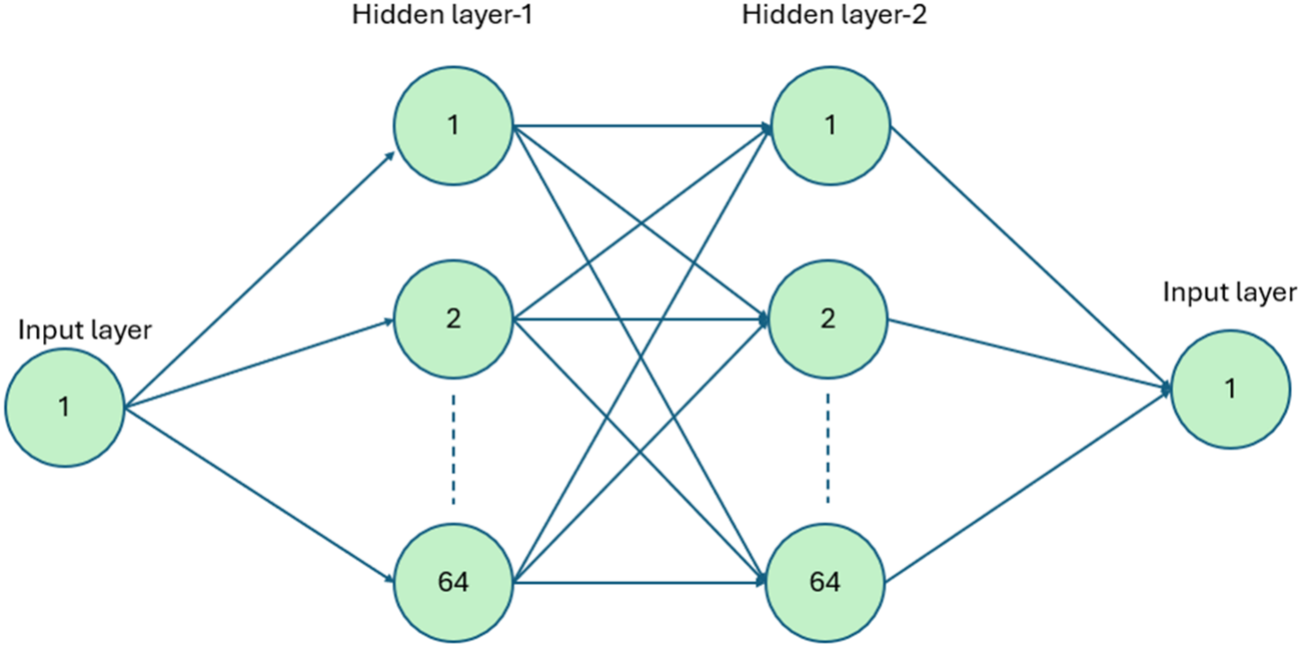
Actor network architecture.

##### 2.1.1.6 Policy Structure

RL methods use neural networks to accurately reflect the policy that the agent is implementing.^13^ Given the strong connection between the policy structure and the RL method, it is impossible to identify the policy structure without simultaneously choosing the RL algorithm. Figure 4 shows the discussed policy structure.

**Figure 4:**
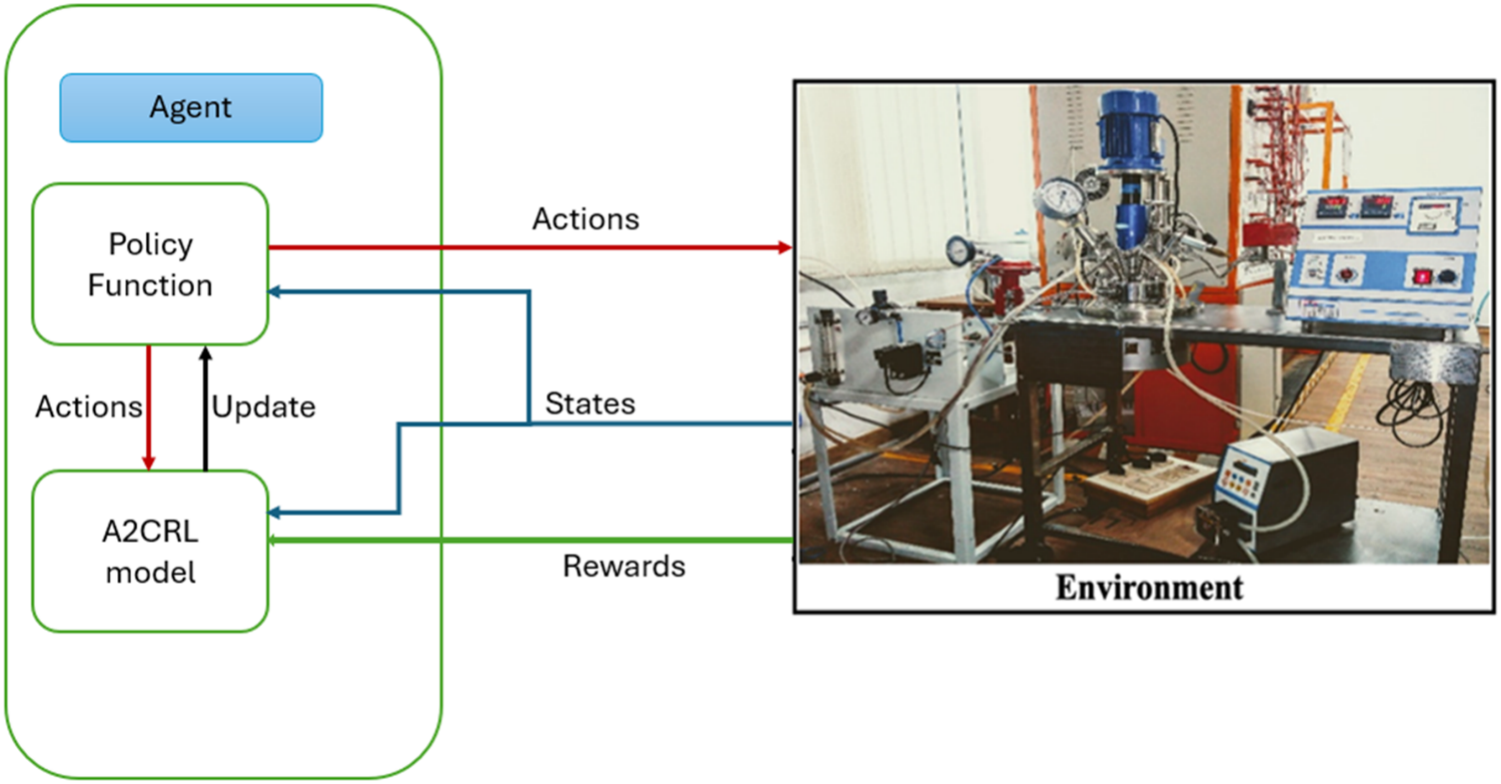
Schematic of policy structure with batch reactor environment.

##### 2.1.1.7 Value Function

The value function of a state represents the expected sum of rewards that an agent will receive in the future, starting with that state. The RL algorithm estimates the value function based on rewards. Rewards assess the immediate, inherent appeal of environmental conditions, while values evaluate the long-term desirability of conditions, including the subsequent states and rewards that may be accessible.^11^

##### 2.1.1.8 Training

In RL, training entails iteratively improving an agent’s performance by allowing it to interact with the environment. The agent follows a policy and receives rewards. The primary goal of training is to optimize the policy so that the agent can maximize the cumulative reward. In several episodes, the agent experiments with a variety of techniques and learns from the results. The computationally expensive training phase requires data and time to produce excellent results.^14,15^

##### 2.1.1.9 Deployment

In the field of RL, the trained agent executes its assigned tasks in the actual physical world or specific environment, as illustrated in Figure 5. This agent adheres to the policies it acquired during its training. During deployment, the agent is required to make instantaneous decisions and potentially react to minor fluctuations in the environment. The process entails thorough monitoring and evaluation to confirm that the agent’s performance aligns with expectations and to implement any necessary enhancements. In order to ensure that the RL model provides value in real-world scenarios without any unforeseen repercussions, the deployment process needs to consider scalability, efficiency, and safety.^14^

**Figure 5:**
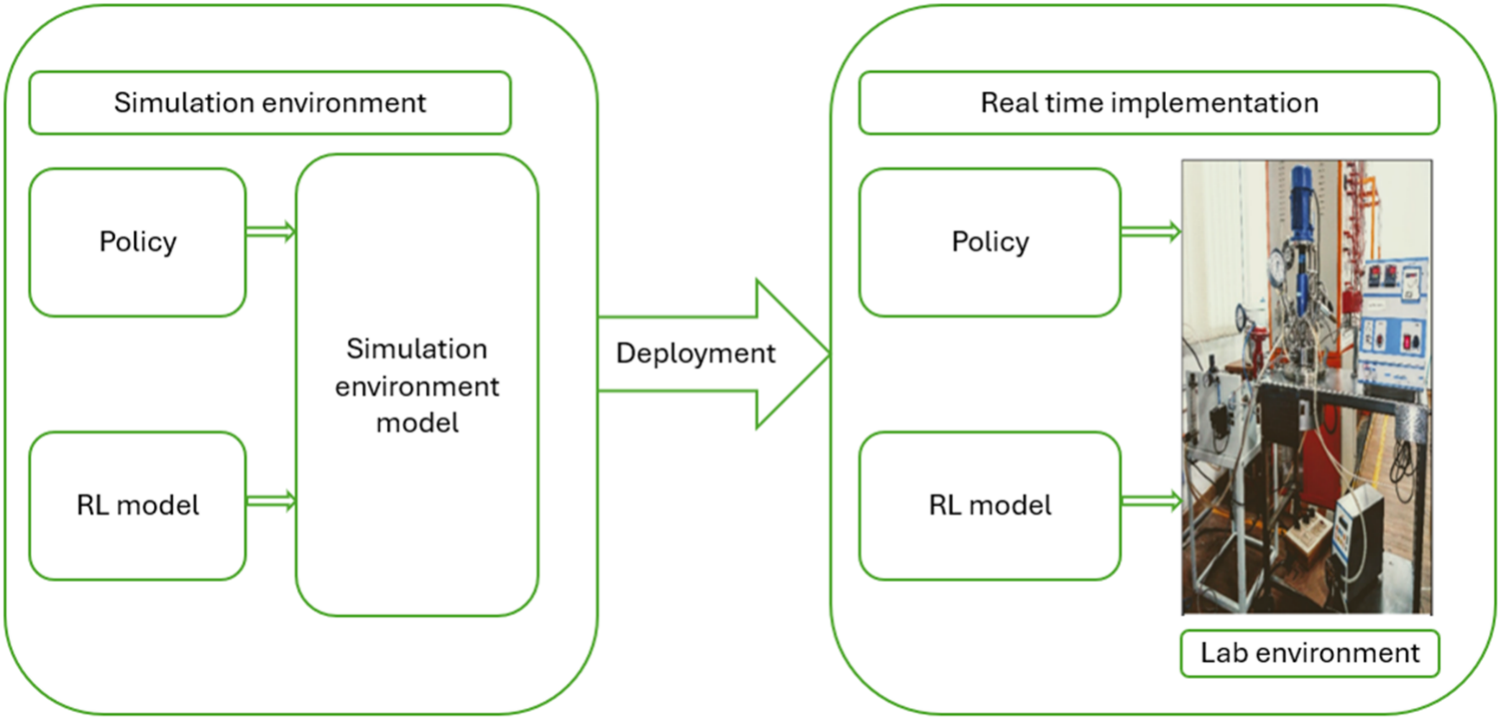
Deployment of A2CRL algorithm on lab scale batch reactor.

#### 2.1.2 Markov Decision Process (MDP)

A Markov Decision Process (MDP) is a stochastic process that can be either discrete time or continuous time. The agent, actions, states (both current and future), the policy (which specifies the action to take), the reward associated with the transition, and the probability of transitioning and changing states are all key components of an MDP. The main challenge posed by MDPs is to determine the optimal policy for the agent based on its current state. The agent is responsible for making decisions and carrying out actions. As the agent transitions between states, the system operates in an environment that specifies its states. The MDP provides a method by which states and agents’ actions lead to transitions to other states. In addition, the agent receives a reward that is proportional to the activity it does and the state it reaches. Based on the agent’s current state, the policy for the MDP determines the action that the agent will take. Figure 6 illustrates the agent-environment interaction. Figure 7 illustrates the real-time implementation of the agent in a BRPP. The MDP model is based on the Markov property. This property says that it is possible to determine the next state purely based on the current state, which contains all the necessary information from the previous states. MDPs are employed to address a diverse range of optimization and control challenges. Employing a range of RL approaches can overcome these issues.^11,16,17^

**Figure 6:**
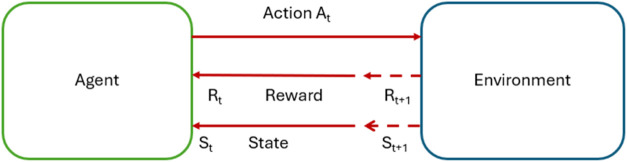
Agent–environment interaction in a Markov decision process.

**Figure 7:**
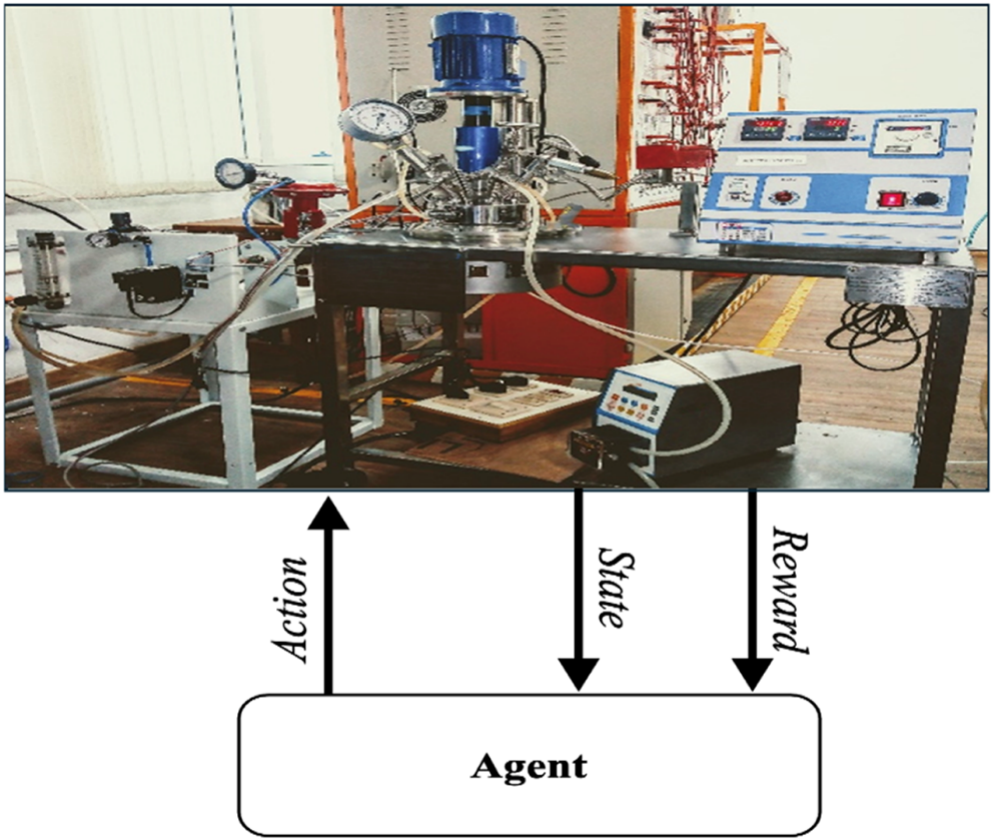
Agent–environment interaction in real time.

In RL, dynamic programming (DP) is a collection of algorithms designed to identify the most efficient strategies. These algorithms identify strategies by assuming a customized MDP environment, a perfect representation of the environment. DP algorithms are particularly well-suited for optimization challenges because they explicitly design to assess previously solved subproblems and combine their solutions to produce the ideal solution for the current problem. This is because they were developed to address optimization issues.^11^

#### 2.1.3 Reinforcement Learning on Control Problems

Reinforcement learning (RL) provides promising results for finding optimal controllers for systems with nonlinear, potentially stochastic dynamics that are either unknown or extremely unpredictable. With an increasing demand for complex nonlinear control systems, the design of optimal controllers using classical methods is also increasing over time. RL is ideal for controlling a nonlinear complex system because it allows it to learn through exploration and adapt to a dynamic environment. There are vast RL algorithms available, such as Temporal Difference, A2C Networks, and Value Function Approximation.^3,18^

#### 2.1.4 The Actor-Critic Framework

Actor-critic methods combine two main networks: the actor network, which suggests actions based on the current policy, and the critic network, which evaluates the action by computing the value function. This dual approach allows for more stable and efficient learning by combining the advantages of policy optimization and value estimation. The actor and critic are neural networks that aim to learn the most optimal behavior through learning. The actor learns the appropriate behaviors by utilizing input from the critic to distinguish between good and bad actions, while the critic learns the value function through receiving rewards in order to effectively evaluate the actor’s actions.^19^

By employing actor-critic approaches, as shown in Figure 8, the agent can use the most advantageous aspects of policy and value function algorithms. These networks can manage both continuous state and action spaces, and they can expedite the learning process when the received reward exhibits a high degree of variance.^20^ In real-time control systems, these methods are particularly beneficial due to their ability to adaptively optimize control policies in the face of changing conditions and requirements. They are used in robotics, autonomous vehicles, and complex real-time systems.

**Figure 8:**
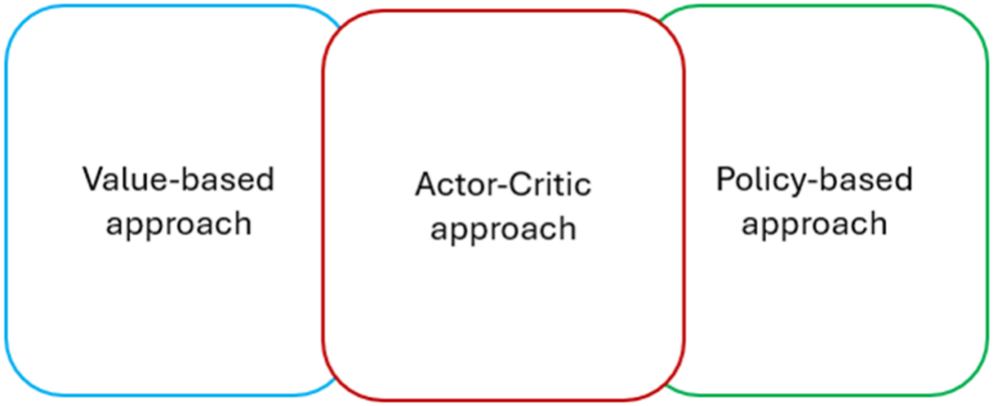
Actor-Critic approaches comparison.

There are various types of actor-critic algorithms designed to improve the efficiency and stability of RL models. The Advantage Actor-Critic (A2C) algorithm utilizes the advantage function to reduce the variance while updating policy parameters (actor weights) and value-function parameters (critic weights).^21^ This approach helps stabilize the learning process by focusing on the relative value of actions rather than their absolute value. On the other hand, the Asynchronous Advantage Actor-Critic (A3C) algorithm builds upon A2C by employing multiple agents that explore different parts of the environment simultaneously. This asynchronous approach not only enhances learning speed but also improves stability, as the diverse experiences gathered by multiple agents help in better approximating the optimal policy and value functions. A3C is particularly effective in complex environments where synchronous updates might lead to slower convergence or instability due to correlated experiences among agents.

An actor is a neural network that gives the agent direct instructions about what actions it should take. Given the current state, the actor network is trying to determine the best possible action. The actor’s objective is to optimize the policy that maximizes expected rewards. In this article, the actor assigns actions to the BR environment. The BR environment is primarily responsible for selecting the best actions.

The critic network represents the value function. This network inputs state observations and outputs the appropriate value. The policy then selects the action with the highest value. Over time, the network will gradually approach a function that accurately predicts the value of any action in any part of the continuous state space. The critic network computes the value function to evaluate the actor’s action. The critic eventually uses the reward earned through the environment to determine the accuracy of its value estimation. The error refers to the difference between the newly estimated value of the previous state and the initial estimate of the previous state obtained from the critic network. The new estimated value is determined by the received rewards and the discounted value of the current state. The error provides the critic network with an idea of whether states turned out good or bad than expected.^22^

### 2.2 Literature Review

Lin et al.^14^ present a novel RL-based MPC framework for discrete-time processes. The framework combines RL with MPC via policy iteration. This framework uses RL for policy evaluation and the MPC as a policy generator. The derived value function serves as the terminal cost in MPC. This improves the resulting policy. This approach eliminates the necessity for the offline design framework concerning terminal costs, auxiliary controllers, and terminal constraints in conventional MPC. Moreover, the RLMPC facilitates an adaptable selection of prediction horizon by removing the terminal requirement, hence significantly diminishing the computing load. Simulation results indicate that RLMPC attains performance comparable to classic MPC in controlling linear systems and demonstrates advantages in managing nonlinear systems.

Small- and medium-sized systems frequently lack a comprehensive dynamic model and instead rely on classical PID controllers, which are unable to ensure consistent set point tracking throughout a process’s evolution. To address this limitation, an NMPC strategy, combined with system identification techniques, has been proposed by Medina–Ramos et al.^23^ This method utilizes a Volterra series-based approximate model, projecting the kernels onto orthogonal basis functions (OBFs) for precise system representation. The NMPC strategy leverages a control technique akin to dynamic matrix control (DMC) and demonstrates superior performance compared to traditional PID controllers, particularly in temperature trajectory tracking. The proposed scheme highlights its potential for facilities where precise temperature control is critical for ensuring high-quality final products, making it a promising alternative to conventional methods in nonlinear systems.

Hassanpour et al.^24^ proposed an offset-free MPC to design an RL-based controller for nonlinear systems without offsets. RL-based controllers can modify their control strategies utilizing real-time data from interactions between the controller and the process. This reduces model maintenance, which is essential in advanced control methods like MPC. Stochastic explorations are essential for an RL agent to identify optimal state-action regions and attain the best policy. This is unfeasible in reality owing to safety and expense. A pretraining method secures online RL controller deployment. Offline RL agent training employs an offset-free model predictive control optimization issue. The RL agent manages the operation in real-time and exhibits performance akin to offset-free MPC. The simulation results illustrate the method’s capacity to manage nonlinearity and operational conditions of the plant induced by unmeasured disturbances. The oscillatory closed-loop responses of the offset-free MPC, caused by differences between the plant and model as well as unmeasured disturbances, can be substantially enhanced by the proposed RL controller.

NMPC is applied to industrial BRs to address safety and productivity limitations resulting from swelling. The catalyst, introduced in incremental stages, generates uncertainty in feeding time, mass, and purity, necessitating online management to manage disruptions. A shrinking horizon optimal control framework, integrated with reaction and hydrodynamic models, is used for real-time optimization. While offline methods optimize the temperature profile for fixed catalyst dosing, the proposed online strategy by Simon et al.^25^ dynamically adjusts to disturbances. This ensures optimal temperature tracking without level swelling, highlighting its effectiveness in maintaining safety and efficiency in BR operations.

Marquez-Ruiz et al.^26^ explore the control of batch processes using MPC and Iterative Learning Control (ILC). Traditional approaches often rely on fixed linear time-invariant (LTI) models, which struggle to capture the nonlinear and time-varying nature of batch processes. The authors propose a novel ML-MPC framework that integrates MPC and ILC with linear parameter-varying (LPV) models to better capture these dynamics. Three estimation methods for model parameters and disturbances are tested through simulations applied to a nonlinear BR. Finally, the ML-MPC approach is demonstrated on an industrial reactive batch distillation column, showing its potential for improving control in time-varying processes.

Zheng and Wu^27^ developed a framework integrating ML models and predictive control schemes to optimize batch crystallization processes. Utilizing a population balance model (PBM) to describe crystal formation dynamics, they trained recurrent neural network (RNN) models on PBM simulations to capture process behavior under diverse conditions. These RNN-based Model Predictive Control (MPC) schemes demonstrated improved computational efficiency and control performance, optimizing crystallization yield, crystal size, and energy consumption while adhering to input constraints.

Wong et al.^28^ explored the use of RNNs for MPC in continuous pharmaceutical manufacturing, focusing on addressing complex dynamics and reaction kinetics. This work emphasized the adoption of data-driven models like RNNs over traditional physics-based models, showcasing effective closed-loop control for a continuous-stirred tank reactor (CSTR). Both studies highlight the significant potential of RNNs in capturing process dynamics and enhancing control strategies in chemical engineering applications.

Meng et al.^29^ explored the application of RNN-LSTM-based MPC in the corn-to-sugar (CTS) process, addressing the challenges posed by complex physical and chemical dynamics. The study emphasized preprocessing and dimensionality reduction of input variables, followed by sensitivity analysis to identify key factors influencing the process. Using these inputs, an RNN-LSTM model was constructed to predict the dextrose equivalent value. The RNN-LSTM served as the predictive model in the MPC system, enabling effective control of the dextrose equivalent under varying set point changes and disturbances in simulation studies. This research demonstrated the potential of RNN-LSTM-based MPC to enhance control precision in the CTS process.

Wijaya et al.^30^ investigated the integration of NNs and RL into control system design, focusing on addressing the limitations of offline NN learning by enabling online adaptation. An LSTM-enabled model-based RL (Mb-RL) approach is utilized to record local system dynamics and adapt to environmental changes. The study utilized an MPC framework as the RL agent, applying a random shooting policy to minimize the control objective function. The proposed method was tested on a nonlinear mass spring damper (NMSD) system with varying inertia parameters, demonstrating effective control over high-oscillating nonlinear systems. The findings highlight the potential of LSTM-enabled Mb-RL in improving the robustness and adaptability of control systems.

Reiter et al.^31^ proposed a hybrid control strategy combining MPC and RL to leverage their complementary advantages. The RL critic approximates the optimal value function, while the actor roll-out provides an initial guess for MPC’s primal variables. A parallel control architecture is introduced, where MPC solves two instances for different initializations: one from the actor roll-out and another from the previous solution’s shifted state. The actor and critic assess the infinite horizon cost of these trajectories, using the control action from the trajectory with the minimal cost at each time step. The proposed method guarantees improved performance over the original RL policy, with an error term that diminishes as the MPC horizon lengthens. This approach is validated through a toy example and an automated driving overtaking scenario, demonstrating its effectiveness in enhancing control performance.

In conclusion, the underlying theory and literature evaluation establish the basis for using RL in batch reactor control. Especially in addressing the complexity and uncertainty inherent in real-time control systems, the review shows the adaptability and efficiency of RL techniques. All the methodologies in the literature review corroborate the model’s performance through simulation; however, this paper conducts validation using experimental data. The proposed architecture is unique in its ability to continuously maximize control actions, balancing policy and value estimations for consistent learning. This research intends to design a strong control strategy for a batch reactor using recent developments in machine learning and RL, potentially going beyond traditional methods like PID controllers. This approach promises enhanced safety, efficiency, and product quality in chemical production processes, aligning with the industry’s evolving demands for advanced control solutions.

The proposed framework is trained using open-loop experimental data

and optimized to dynamically adjust the coolant flow rate, ensuring precise temperature regulation and stability. Compared to existing deep learning-based NMPC implementations, the proposed actor-critic methodology enhances NMPC controller performance by balancing prediction accuracy and real-time computational efficiency. Section 3 delineates the suggested model comprehensively.

## 3 Proposed Methodology for the Model Validation

This section highlights the evolution of the RNN model, its mathematical formulation, BRs, batch polymerization, the A2C framework for NMPC parameter optimization, the A2C algorithm for the NMPC parameter optimization workflow, and the integration of the RNN model with MPC.

### 3.1 Development of RNN Model

Recurrent neural networks (RNNs) are proficient in modeling complex and nonlinear interactions between input and output data, making them particularly suitable for time-series data and nonlinear problems. Leveraging their ability to process sequential information, RNNs can retain contextual information from past inputs,^32^ which is essential for understanding the temporal dependencies in BR dynamics.

This paper develops the RNN model utilizing data obtained from comprehensive open-loop simulations of the BR, encompassing variables such as reactor temperature (*T_r_*), coolant flow rate (*F_c_*), jacket temperature (*T_j_*), and heater current (*H*). The *T_r_* is considered the state *x*, while the *F_c_* serves as the control input *u*. A total of 10,300 examples were generated with a fine time step during the simulations.

To handle the inherent complexity of accurately describing the dynamic changes in the BR, the time-series data is transformed into dynamic data sets using an extended sliding window approach. This transformation allows the model to effectively leverage past information for predicting future states. The data set is subsequently divided into training and test sets for the purposes of model training and evaluation.

The RNN is configured as a multiple-input, single-output system to predict the *T*_r_. Specifically, the model is trained to forecast *x*(*t* + 1) based on the current and past reactor temperatures *x* (*t* = 0, 1,···,*l*) and coolant flow *u*(*t* = 0, 1,···, *l*). The time lag *l* is obtained from experimental optimization, where values from 10 to 70 were tested, and *l* = 50 was found to yield the lowest root mean squared error (RMSE) on the validation set. This value effectively balances model complexity and accuracy by capturing long-term temporal dependencies without overfitting. By adjusting *F_c_* based on *T_r_*, the model ensures efficient regulation of the heat generated by the exothermic reaction in the BR.

#### 3.1.1 Structure of RNN Model

The Recurrent Neural Network (RNN) model developed for predicting reactor states is implemented in PyTorch. This model effectively captures temporal dependencies in the BR dynamics using sequential data and is structured as a multiple-input, single-output system. A detailed description of the architecture and its mathematical formulation are given in Sections 3.1.2 and 3.1.3.

#### 3.1.2 Input-Output Structure

The RNN model is structured as a multiple-input, single-output system to predict the *T*_r_ at the next time step *x*(*t* + 1), leveraging sequential data on current and past states *x*(*t* = 0, 1,···, *l*) and control inputs *u*(*t* = 0, 1,···, *l*). The architecture includes: an *input layer* for processing multivariate time-series data, a *recurrent layer* for learning temporal relationships, and a *fully connected output layer* for predicting the *T*_r_. The system utilizes a sequence-to-single-output approach where predictions are based on the hidden state of the final time step.

#### 3.1.3 Mathematical Formulation of the RNN

This section derives the mathematical formulation of the RNN model’s input-output structure, recurrent layer dynamics, and output layer.^26^

##### 3.1.3.1 Input Representation

Equation 2 represents the vector input to the model at each time step *t*.

2 $$x_{t}=\left[T_{r}\left(t\right),F_{t}\left(t\right)\right]\in R^{n_{features}}$$

where *n*_features_ is the number of input features.

The input sequence over a time horizon (*l*) is given in eq 3

3 $$X=\left\{x_{1},x_{2},\cdot \cdot \cdot x_{l}\right\}$$

##### 3.1.3.2 Input-Output Structure

The system is formulated as shown in eq 4

4 $$y_{t}=f\left(x_{1},x_{2},\cdot \cdot \cdot x_{l},u_{1},u_{2},\cdot \cdot \cdot u_{l}\right)$$

where *f* is a nonlinear mapping learned by the RNN.

##### 3.1.3.3 Recurrent Layer Dynamics

The recurrent layer computes a hidden state *h*_*t*_ using eq 5 at each time step based on the current input *x*_*t*_ and the previous hidden state *h*_*t*–1_.

5 $$h_{t}=f\left(W_{h}x_{t}+U_{h}h_{t-1}+b_{h}\right)$$

where

- *W*_*h*_ ∈ *R* ^*n*_hidden_ × *n*_features_^: Input-to-hidden weight matrix
- *U*_*h*_ ∈ *R* ^*n*_hidden_ × *n*_hidden_^: Hidden-to-hidden weight matrix
- *b*_*h*_ ∈ *R* ^*n*_hidden_^: Bias vector
- *f*: Activation function (ReLU).

Equation 6 gives the output of the recurrent layer across all time steps as a sequence of hidden states

6 $$H=\left\{h_{1},h_{2},\cdot \cdot \cdot h_{l}\right\}$$

where *h*_*t*_ ∈ *R* ^*n*_hidden_^ encapsulates the temporal information up to time *t*.

##### 3.1.3.4 Output Layer

The output layer uses the hidden state of the final time step *h*_*l*_ to predict the next state *x* (*t* + 1). The transformation is defined as given in eq 7.

7 $$y=W_{o}h_{l}+b_{o}$$

where

- *W*_*o*_ ϵ *R* ^*n*_hidden_ × *n*_features_^: Weight matrix of the fully connected layer
- *b*_0_ ∈ *R* ^*n*_output_^: Bias vector
- *y* ∈ *R* ^*n*_output_^: Predicted state.

##### 3.1.3.5 Training Objective

The model is trained to minimize the mean squared error (MSE) between the predicted reactor temperature (*y*) and the actual temperature (*ŷ*) from simulation data. The MSE to calculate the loss is given in eq 8.

8 $$\mathrm{MSE}\;\mathrm{Loss}=\frac{1}{N}\sum_{i=1}^{N}{\left(y^{\left(i\right)}-{ŷ}^{\left(i\right)}\right)}^{2}$$

where *N* is the number of training samples. Figure 9 shows the MSE loss for the predicted and actual *T*_r_. Figure 10 shows the RNN model fit for the BR open loop data.

**Figure 9:**
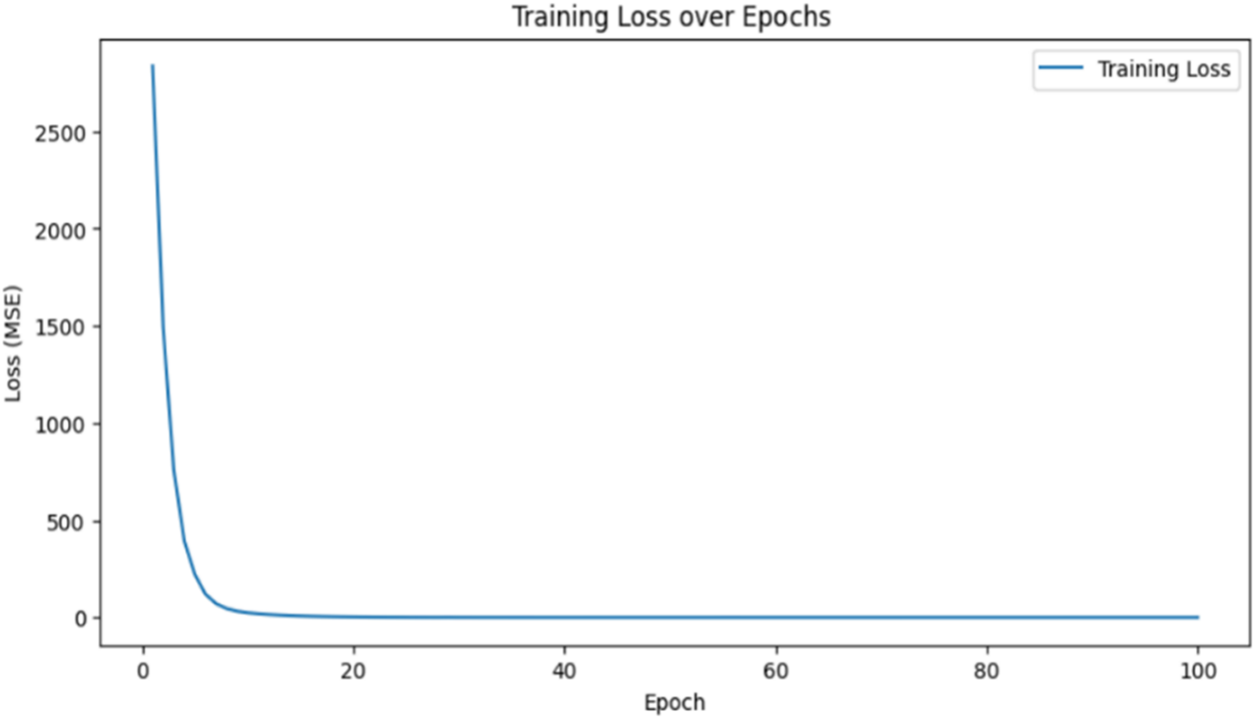
Plot showing the mean square loss for the predicted Vs actual reactor temperature.

**Figure 10:**
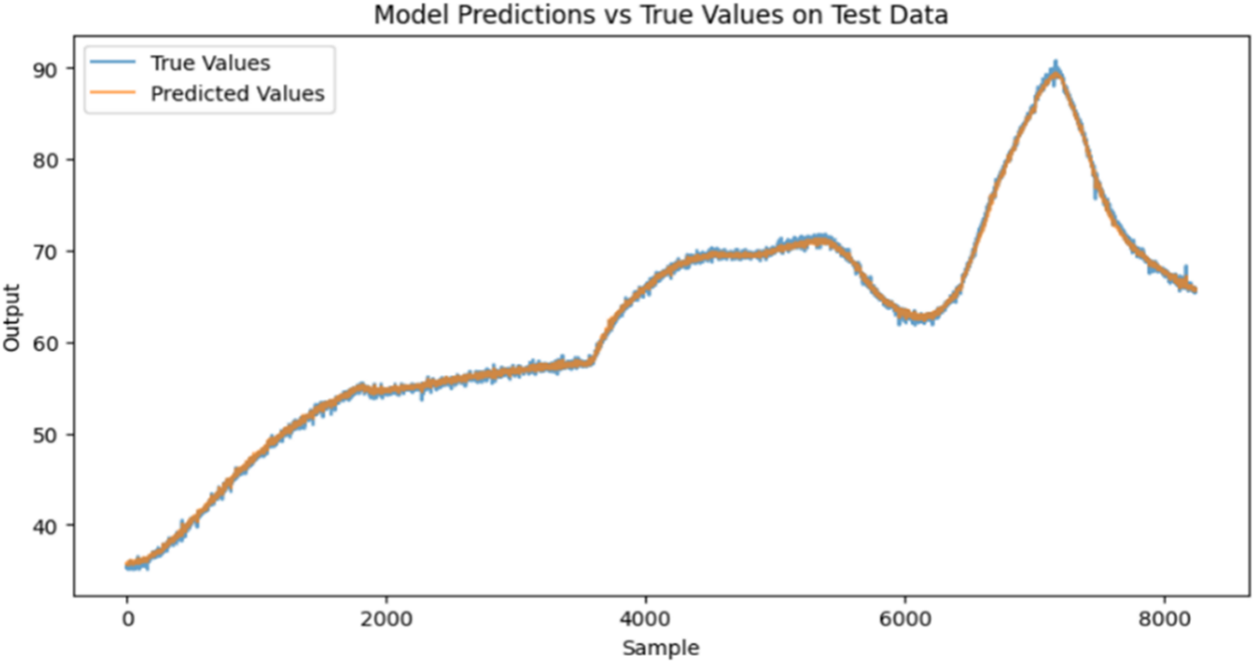
Recurrent neural network model fit of batch reactor open loop data.

### 3.2 Nonlinear Model Predictive Controller (NMPC) Design

The NMPC model forecasts over a limited prediction horizon spanning several timesteps, focusing on optimizing the *F_c_* to maintain the desired *T_r_*. It ensures the efficient operation of the BR by determining the appropriate coolant flow outlet. By leveraging past *T_r_* data, *F_c_*, and predictions from the RNN model, the NMPC prescribes the required coolant flow adjustments.

To manage the manipulated variable (*u*), a soft constraint approach is adopted. Slack variables, integrated into the objective function to be minimized, provide flexibility by relaxing the imposed limits. For a time step *t*, with a fixed prediction horizon (*N_p_*) and a control horizon (*N_c_*), the scheduling framework is formulated as given in eq 9.

9 $$J=\min_{u\left(k|k\right)\cdot \cdot \cdot u\left(k+\mathrm{Nc}-1|k\right)}\sum_{j=1}^{Np}W_{E}\left(E^{2}\left(k+j|k\right)\right)+\sum_{j=0}^{Nc-1}W_{u}\left(\Delta u^{2}\left(k+j|k\right)\right)$$

where *E*(*k* + *j*|*k*) *and* Δ*u*(*k* + *j*|*k*) are calculated using eqs 10 and 11 respectively.

10 $$E\;\left(k+j|k\right)=y_{sp}\left(k+j|k\right)-\hat{y_{c}}\left(k+j|k\right),\forall j=1,2,\cdot \cdot \cdot N_{p}$$

11 $$\Delta u\;\left(k+j|k\right)=u\left(k+j|k\right)-u\left(k+j-1|k\right),\forall j=0,1\cdot \cdot \cdot N_{c}-1$$

with subject to constraints

$$u_{\min}\leq u\left(k+j|k\right)\leq u_{\max},\forall j=0,1,2,\cdot \cdot \cdot N_{c}-1$$

$$\begin{array}{l} \Delta u\left(k+N_{c}|k\right)=\Delta u\left(k+N_{c}+1|k\right) \end{array}=\cdot \cdot \cdot \Delta u\left(k+N_{c}+1|k\right)=0$$

Equations 9 to 11 delineate the NMPC objective function and control constraints. References 1 and 33 cite the author’s previously published works on NMPC with numerical control restrictions to enhance reader comprehension.

#### 3.2.1 Batch Polymerization

Batch polymerization involves the dynamic interplay of the reactor, jacket energy balance, and reaction kinetics equations. In this study, the acrylamide polymerization reaction is considered, with the concentrations of the initiator ammonium persulfate [*I*] and the monomer acrylamide [*M*] estimated using appropriate approximations as given in eqs 12 and 13.

12 $$\frac{d\left[I\right]}{dt}=A_{d}\left[I\right]e^{\left(-E_{d}/R\left(T_{r}+273.15\right)\right)}$$

13 $$\frac{d\left[M\right]}{dt}=-A_{p}{\left[I\right]}^{\varepsilon }{\left[M\right]}^{\theta }e^{\left(-E_{p}/R\left(T_{r}+273.15\right)\right)}$$

The dynamics of the reactor temperature *T*_*r*_ and the jacket temperature *T*_*j*_ are calculated using eqs 14 and[Disp-formula eq15].

14 $$m_{r}c_{\mathrm{pr}}\frac{dT_{r}}{dt}=Q_{r}-\mathrm{UA}\left(T_{r}-T_{j}\right)+Q_{h}+Q_{s}-Q_{\mathrm{loss}}$$

15 $$m_{j}c_{\mathrm{pj}}\frac{dT_{j}}{dt}=\mathrm{UA}\left(T_{r}-T_{j}\right)-F_{c}c_{\mathrm{pc}}\left(T_{j}-T_{c}\right)$$

Here the values for *Q*_*r*_, *Q*_*s*_, *Q*_loss_, *m*_*r*_ *c*_*pr*_, and *m*_*j*_ *c*_*pj*_ are calculated using eqs 16–20.

16 $$Q_{r}=\frac{d\left[M\right]}{dt}V\Delta H_{p}$$

17 $$Q_{s}=p_{0}\rho n^{3}d_{5}$$

18 $$Q_{\mathrm{loss}}=\alpha {\left(T_{r}-T_{\mathrm{amb}}\right)}^{\beta }$$

19 $$m_{r}c_{\mathrm{pr}}=\sum_{i=1}^{6}m_{i}c_{\mathrm{pi}}$$

20 $$m_{j}c_{\mathrm{pj}}=\sum_{i=7}^{8}m_{i}c_{\mathrm{pi}}$$

The heat loss coefficients α and β are evaluated using a least-squares approach, and the overall heat transfer coefficient *U* is computed based on the BR’s time constant.

### 3.3 Actor-Critic Framework for NMPC Parameter Optimization

The A2C algorithm is a reinforcement learning method that integrates the benefits of policy-based and value-based approaches.^34^ It is particularly suited for solving continuous control problems, such as fine-tuning the parameters of the objective functions in NMPC for BRs. This section outlines the A2C framework and its components, emphasizing its mathematical foundation and relevance to the NMPC system.

#### 3.3.1 Overview of the A2C Architecture

The A2C framework consists of two main components:

1. *Actor*: The policy network responsible for selecting actions (e.g., control parameters like *R*_1_ and *R*_2_ in NMPC). The actor outputs a probabilistic or deterministic policy, π_θ_(*a*|*s*), which defines the likelihood of taking an action *a* in a given state *s*.
2. *Critic*: The value network responsible for evaluating the quality of actions taken by the actor. It estimates the expected return (cumulative reward *V*_ϕ_(*s*)), for a given state *s*, guiding the actor to improve its policy.

The interaction between the actor and the critic enables efficient learning and fine-tuning of parameters.

#### 3.3.2 Problem Formulation in the NMPC Context

For a BR system controlled via NMPC, the A2C algorithm optimizes the parameters of the objective function by learning from the state transitions and feedback rewards. Let: *s*_*t*_ represent the state of the reactor at time *t*, including *T*_*r*_, *F*_*c*_, and other relevant dynamics. *a*_*t*_ = [*R*_1_, *R*_2_] represents the control parameters (e.g., weighting factors or reference values) output by the actor. *r*_*t*_ denotes the reward received after applying action *a*_*t*_ in state *s*_*t*_, which reflects the performance of the NMPC system, such as minimizing temperature deviations or optimizing energy efficiency.

The goal is to maximize the cumulative reward *J*(π_θ_) given in eq 21.

21 $$J\left(\pi_{\theta }\right)=E_{\pi_{\theta }}\left[\sum_{t=0}^{\infty }\gamma^{t}r_{t}\right]$$

where γ ∈ [0,1] is the discount factor.

##### 3.3.2.1 Actor Network–Policy Formulation and Parameter Scaling

The actor defines the policy π_θ_(*a*|*s*), parametrized by θ, and outputs the control parameters *R*_1_ and *R*_2_. The policy is updated using the deterministic policy gradient theorem given in eq 22.

22 $$\nabla_{\theta }J\left(\pi_{\theta }\right)=E_{s∼p^{\pi },a∼\pi_{\theta }}\left[\nabla_{\theta }\pi_{\theta }\left(a|s\right)\nabla_{a}Q^{\pi }\left(s,a\right)\right]$$

where:

- *Q* ^π^ (s,a) is the action-value function, estimated by the critic.
- ∇_θ_π_θ_(*a*|*s*) represents the gradient of the policy with respect to its parameters.

The actor produces scaled outputs *R*_1_ and *R*_2_ using eqs 23 and 24.

23 $$R_{1}=\sigma \left(r_{1,\mathrm{raw}}\right).\nabla_{R_{1}}+\mu R_{1}$$

24 $$R_{2}=\sigma \left(r_{2,\mathrm{raw}}\right).\nabla_{R_{2}}+\mu R_{2}$$

Here, *R*_1_ penalizes temperature tracking error, ensuring the reactor follows the reference trajectory. *R*_2_ penalizes excessive fluctuations in coolant flow to avoid aggressive control actions. σ is the sigmoid activation function, which ensures that the values remain within a stable range. ∇_*R*_1__, ∇_*R*_2__ define the scaling range, corresponding to

- *R*_1_ scaled between 15, 50
- *R*_2_ scaled between 5, 1
- μ*R*_1_, μ*R*_2_ are offset values, ensuring an appropriate initialization for NMPC.

The sigmoid activation function σ­(*x*) restricts the raw outputs *r*_1,raw_ and *r*_2,raw_ within a normalized range [0,1] before applying the scaling transformation. These weights *R*_1_ and *R*_2_ are dynamically adjusted by the ATN using reinforcement learning. The actor learns an optimal trade-off between tracking accuracy and control smoothness.

##### 3.3.2.2 Reward Structure

The reward function is derived from the negative optimal cost obtained from the NMPC cost function. This approach ensures that minimizing cost directly aligns with maximizing the RL reward, thus guiding optimal control actions. The formulation is given in eq 25.

where:

25 $$r_{t}=-J_{NMPC}$$

- *J*_NMPC_ is the cost function of the NMPC optimization problem, accounting for tracking errors and control effort.
- A lower NMPC cost corresponds to a higher reward, reinforcing optimal tuning of NMPC parameters.

##### 3.3.2.3 Critic Network

The critic evaluates the quality of the actor’s policy by estimating the state-value function *V*_⌀_(*s*) or the action-value function *Q* ^π^(*s*, *a*) using eq 26.

26 $$Q^{\pi }\left(s,a\right)=E_{s^{\prime }∼p^{\pi }}Q^{\pi }\left(s,a\right)=E_{s^{\prime }∼p^{\pi }}[r\left(s,a\right)+\gamma V_{\phi }\left(s^{\prime }\right)$$

The critic’s parameters ϕ are updated by minimizing the temporal difference (TD) error using eq 27.

27 $$L_{\mathrm{critic}}\left(\phi \right)=E_{\left(s,a,r,s^{\prime }\right)}\left[{\left(Q^{\pi }\left(s,a\right)-\mathrm{Q̂}\left(s,a\right)\right)}^{2}\right]$$

here

$$\mathrm{Q̂}\left(s,a\right)=r\left(s,a\right)+\gamma V_{\phi }\left(s^{\prime }\right)$$

is the TD target.

The critic guides the actor by providing gradients of *Q* ^π^(*s*, *a*) with respect to a.

##### 3.3.2.4 Ornstein–Uhlenbeck Noise for Exploration

In continuous action spaces, the A2C algorithm often employs exploration noise to ensure sufficient exploration of the state-action space. In this implementation, Ornstein–Uhlenbeck (OU) noise is used, which is defined as given in eq 28.

28 $$x_{t+1}=x_{t}+\theta \left(\mu -x_{t}\right)+\sigma \eta_{t}$$

here θ controls the mean reversion speed, μ is the mean of the noise process, σ determines the noise amplitude, $\eta_{t}∼N\left(0,1\right)$ is Gaussian noise.

This noise facilitates exploration by adding temporally correlated variations to the action space. In this implementation, the Ornstein–Uhlenbeck (OU) noise parameters are carefully chosen to balance exploration and stability in the A2C reinforcement learning framework. The mean reversion speed θ = 7 ensures that the noise quickly returns to its mean value μ, preventing excessive deviation and maintaining stable exploration. The mean value μ = 10 acts as the baseline around which the noise fluctuates, influencing the scale of control parameter adjustments for *R*_1_ and *R*_2_. Additionally, the noise amplitude σ = 10 introduces high variability in the action space, promoting diverse exploration and preventing premature convergence to suboptimal policies. These values were empirically tuned through an ablation study, in which each parameter was varied while observing its effect on convergence speed, variance in policy updates, and closed-loop control stability. The chosen configuration demonstrated the fastest convergence and minimized oscillations in reward trends. Together, these parameters enable the RL agent to explore a wide range of NMPC weight values while ensuring that the learning process remains stable and responsive to reactor dynamics.

##### 3.3.2.5 Actor-Critic Algorithm for NMPC Parameter Optimization Workflow

The CRN evaluates the control performance and updates the ATN, refining *R*_1_ and *R*_2_ over time. A flowchart for the A2C algorithm for the NMPC parameter optimization is given in Figure 11.

**Figure 11:**
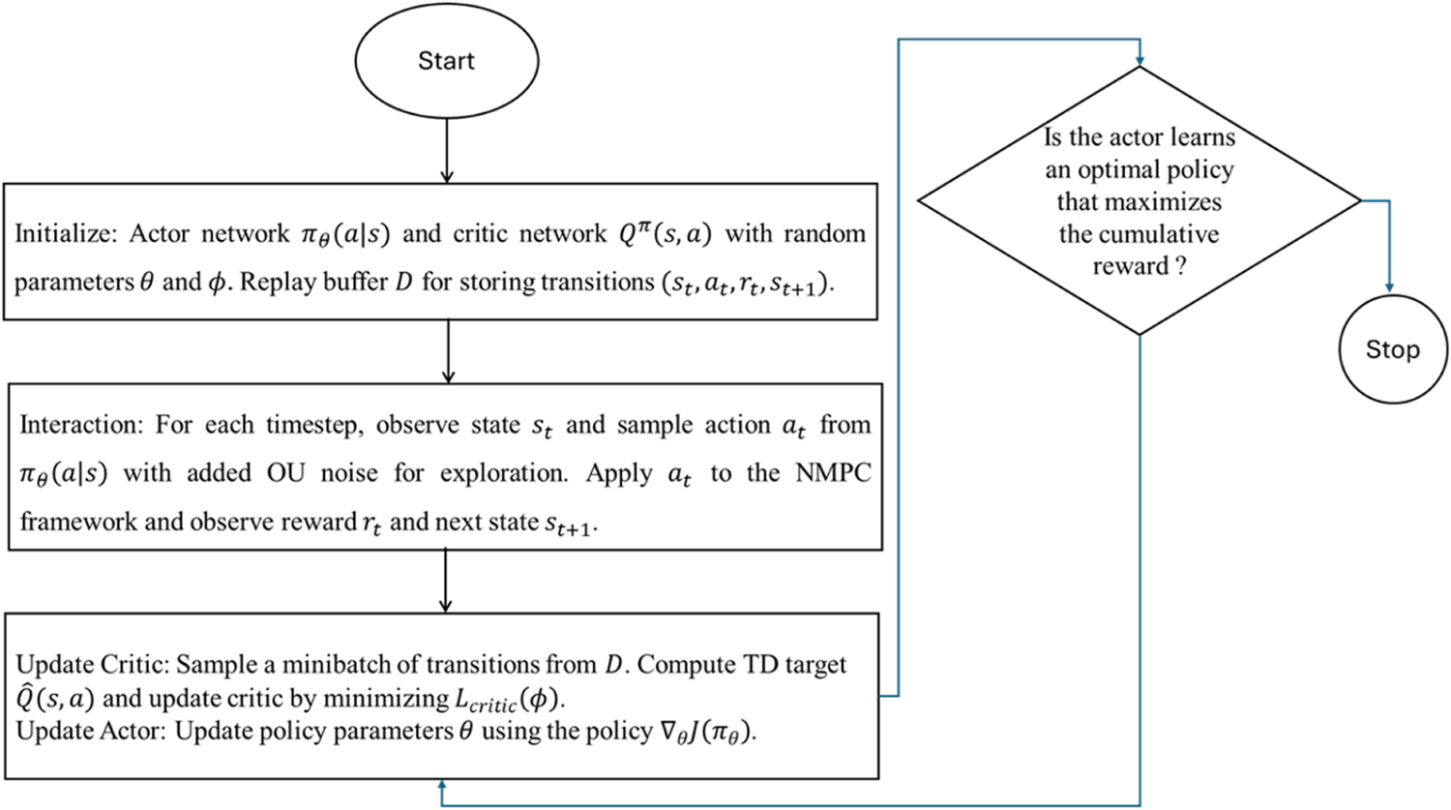
Flowchart for the Actor-Critical algorithm for the NMPC parameter optimization.

Figure 12 illustrates the block diagram of the comprehensive framework. Initially, open-loop data gathering was conducted from the batch reactor. The subsequent phase involved the modeling and validation of the RNN. The subsequent phase involved the implementation of RL-NMPC design utilizing RNN model predictions. The code was subsequently downloaded into the Jetson Orin. The subsequent phase involved the scaling of signals. The real-time validation of RL-NMPC has been successfully accomplished.

**Figure 12:**
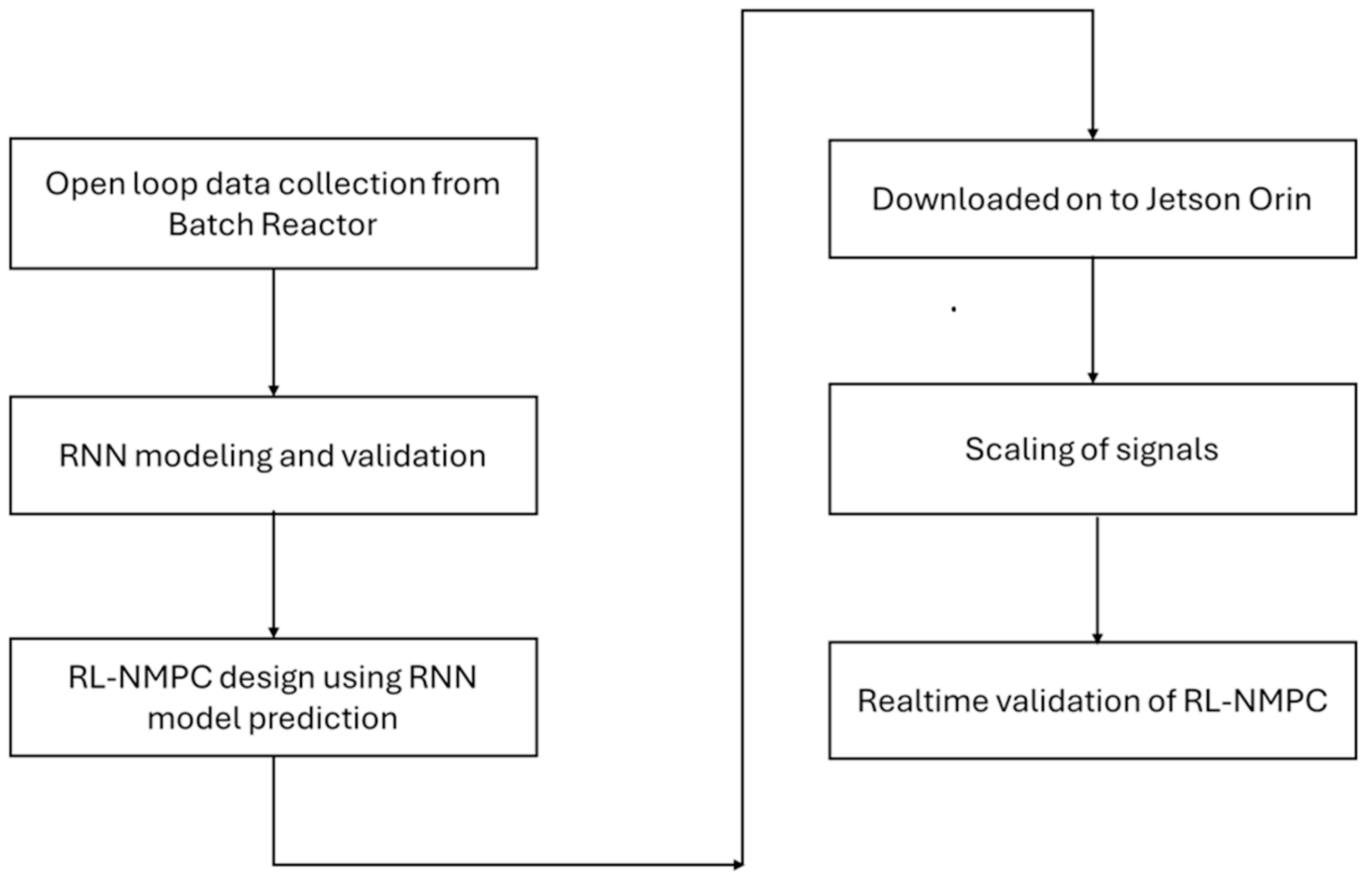
Block diagram for the overall framework.

The A2C algorithm for NMPC parameter optimization follows these steps:
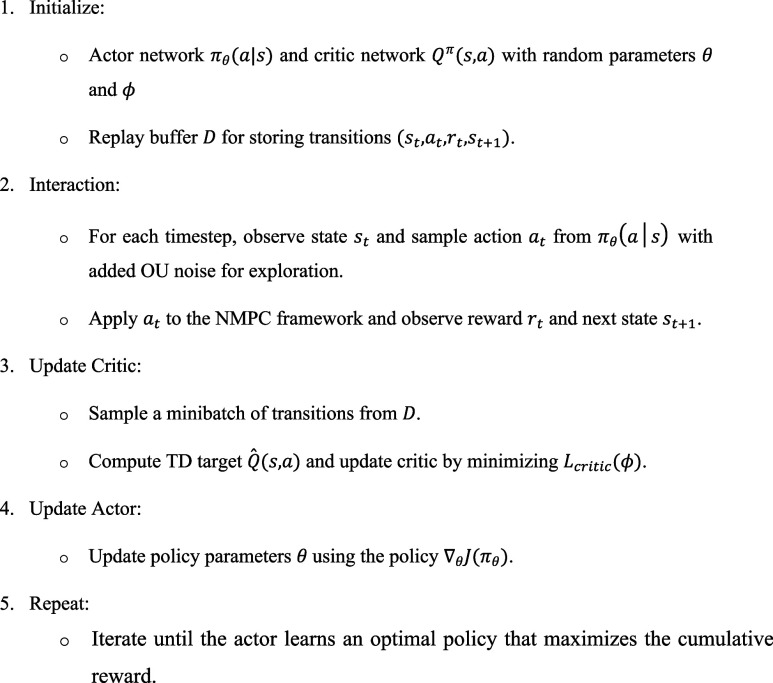


By integrating the A2C reinforcement learning algorithm with Nonlinear Model Predictive Control (NMPC) for the jacketed batch reactor (BR):

- The ATN dynamically adjusts the NMPC weighting parameters (*R*_1_, *R*_2_) in real-time, optimizing the balance between temperature set point tracking and coolant flow regulation. The *F*_*c*_ is the primary manipulated variable that counteracts exothermic reaction heat, ensuring reactor stability. The *T*_*r*_ serves as the key process state, influencing reaction kinetics and product yield.
- The CRN evaluates the long-term impact of these adjustments, ensuring improved reactor stability, reduced control effort, and energy efficiency.
- The addition of Ornstein–Uhlenbeck (OU) noise facilitates exploration, allowing the RL agent to handle process uncertainties, exothermic reaction dynamics, and nonlinearities in the reactor system.

This RL-NMPC framework enables real-time adaptive control of *T*_*r*_ on the Jetson Orin platform, ensuring smooth control actions while mitigating oscillations and enhancing process efficiency.

Given that it is a nonsteady-state temperature profile, the initial conditions remain constant. A nonlinear temperature profile is employed as a reference trajectory for tracking with RL-NMPC. This study does not examine the disturbance component; nevertheless, the authors are developing a method for self-correction of the temperature profile in the presence of disturbances. Soon, authors will be able to write a paper featuring self-correction of the temperature profile in the presence of uncertainty.

### 3.4 Combining RNN Model with MPC

Model Predictive Control (MPC) is a robust control strategy that relies on a dynamic system model to predict future states and optimize control actions over a defined horizon while satisfying system constraints.^35^ Traditionally, these system models are derived from first-principles equations, such as energy balance and reaction kinetics for BRs. However, deriving and solving such models can be computationally expensive and prone to inaccuracies due to unmodeled dynamics or parameter uncertainties, posing challenges for real-time applications.^36^ To address these issues, ML models, particularly recurrent neural networks (RNNs), offer a data-driven alternative. RNNs can learn complex, nonlinear system dynamics directly from data, eliminating the need for explicit physical modeling.^37^ They provide faster predictions and improved accuracy by capturing time dependencies and intricate nonlinearities, making them ideal for integration with MPC. By replacing the traditional prediction model in MPC with an RNN, the combined framework leverages the predictive power of deep learning while maintaining the robustness and constraint-handling capabilities of MPC.^38^ In this study, the methodology from ref 39 is employed to merge a PyTorch-based RNN model with the CasADi framework, enabling efficient implementation and real-time optimization for BR control.^35^

### 3.5 Forecasting Ability of Proposed RNN Model with A2CRL

This article implemented the A2CRL algorithm in Python on a Jetson Orin 8GB Nano board, aiming to control the optimal *T*_*r*_ of the BR system by tracking the reference temperature profile.^40^ The agent is trained in a simulation environment that accurately replicates a BR’s dynamic behavior. This A2CRL approach demonstrated oscillations in controlling the *T*_*r*_ conditions, and it could struggle to constantly maintain the desired *T*_*r*_. Figure 13 illustrates the simulated closed-loop response for BR. Figure 14 illustrates the rate at which coolant flows. The outcome indicates oscillations in the reactor’s temperature compared to the reference trajectory’s temperature. After training the reinforcement model for several episodes, we found that the tracking was not perfect. This motivated us to propose the NMPC framework that tracks the temperature profile in a BR using an A2CRL methodology and an RNN-based approach. The authors would like to note that standard PID control does not adequately address the temperature profile tracking of batch reactors. In the past, authors have applied conventional PID controllers to a batch reactor for temperature trajectory tracking. Due to the dynamic nature of the set point and the process’s nonlinearity, the PID controllers exhibited inadequate control signal variations between 0 and 100 on a continuous basis.^41^ This behavior of control action may result in pneumatic control valve failure or diaphragm rupture. Consequently, the pursuit of sophisticated control algorithms emphasizing seamless control action.

**Figure 13:**
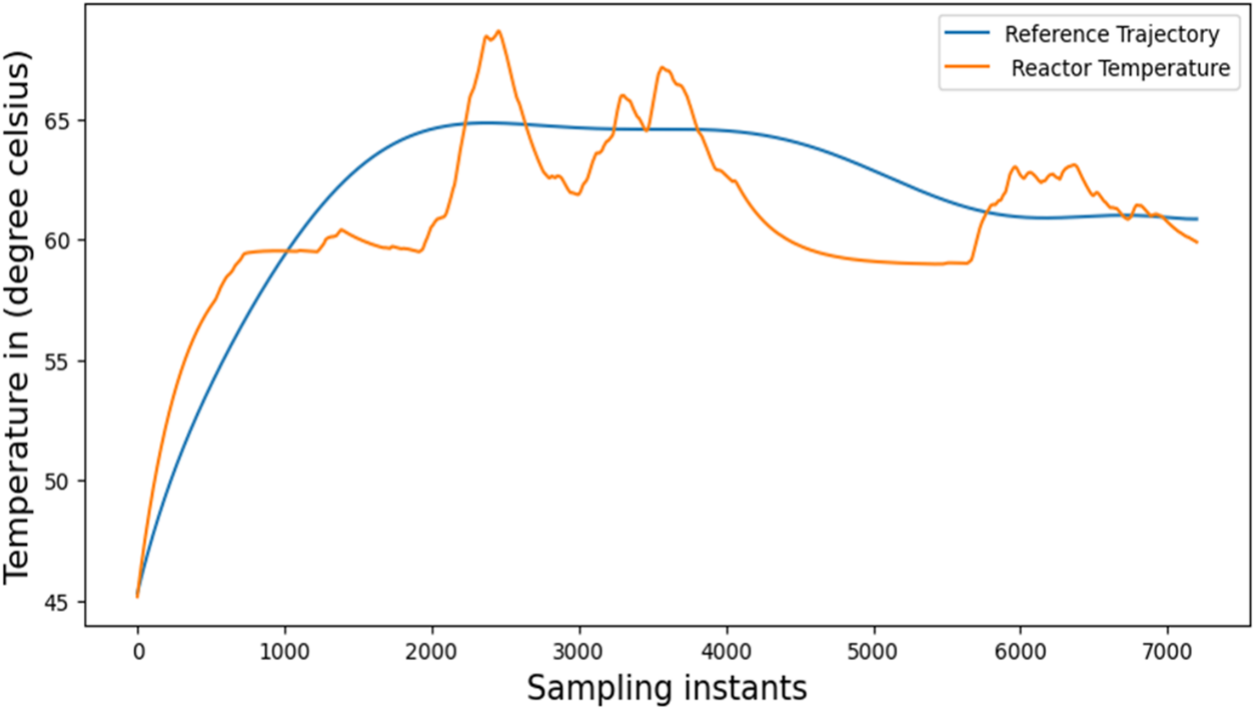
Closed loop trajectory tracking of RL in Simulation.

**Figure 14:**
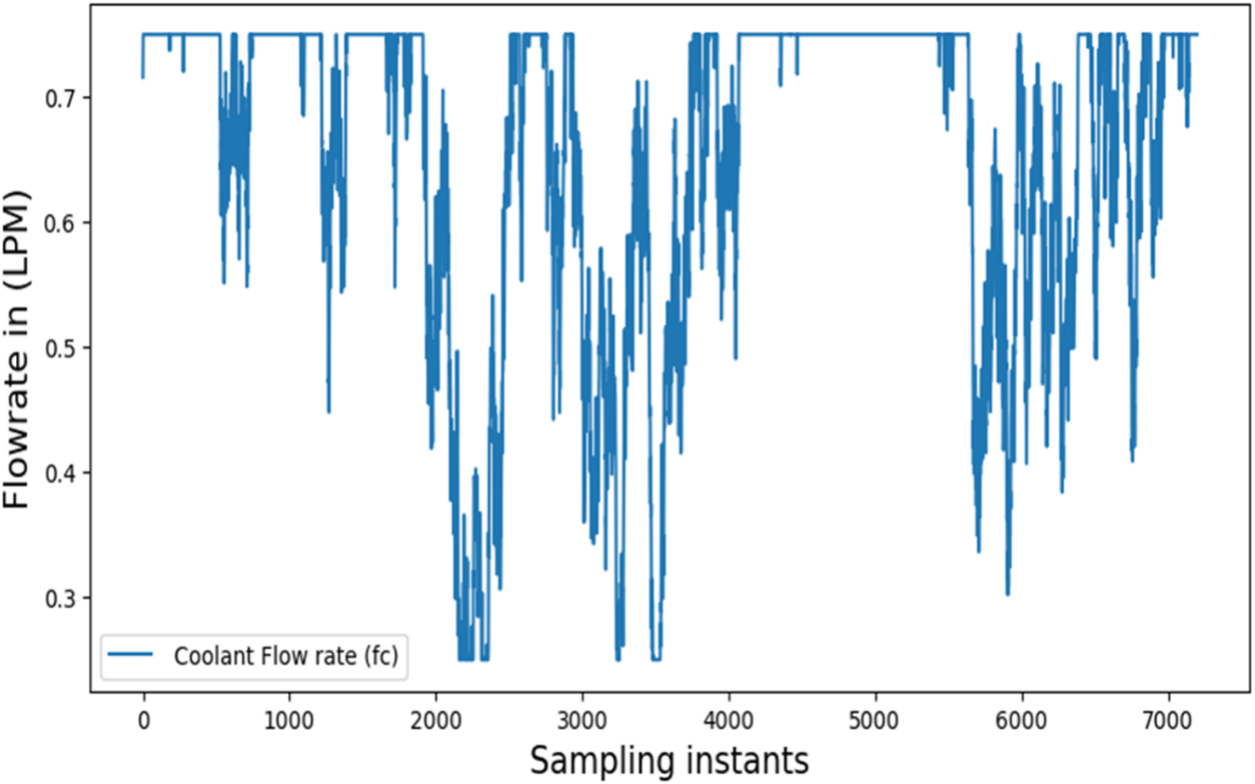
Control signal of RL controller-simulation.

In this article, the open loop *T*_*r*_ data was gathered by varying the *F*_*c*_ while maintaining a constant heater supply, resulting in a data set of 10,300 samples. Since noise and measurement delays were minimal, no additional preprocessing techniques were required. This data set was subsequently utilized to develop the BR model using an RNN.

To assess the forecasting performance of the proposed model, a sequence length *l* of 50 days was employed for training without introducing any lag. The model was trained over 100 epochs using an RNN architecture with 128 hidden units per layer. The ReLU activation function was used implicitly in the RNN layer to introduce nonlinearity and stability. A batch size of 64 was selected to balance computational efficiency and convergence stability. Hyperparameter tuning was performed through an iterative process, where different combinations of learning rates, hidden units, batch sizes, and activation functions were tested. The final configuration was chosen based on empirical validation, with a learning rate of 1e-3 optimized using the Adam optimizer to ensure stable convergence while preventing overfitting. The Mean Squared Error (MSE) loss function was used to measure prediction accuracy. Notably, this configuration reduced the final training MSE from ∼17.05 (with learning rate 1 × 10^–2^, batch size 32) to 0.0009, demonstrating a significant improvement in convergence.

The trained RNN model is used to predict the next *N*_*p*_ states at each time step, which are then used by the NMPC to minimize the cost function. By feeding the weights *W*_*e*_ and *W*_*u*_ from the A2C algorithm, the *F*_*c*_ is predicted by the NMPC. Figure 15 illustrates the tracking of the *T*_*r*_ in relation to the desired trajectory and the control signal of RL-NMPC in simulation. The effectiveness of the proposed approach is further confirmed by the performance measures. Table 1 shows the comparison of performance indices with an Integral MSE of 0.6103, an MAE of 0.6152, and an MSE of 0.8005, the model demonstrates exceptional tracking accuracy and predictive ability.

**Figure 15:**
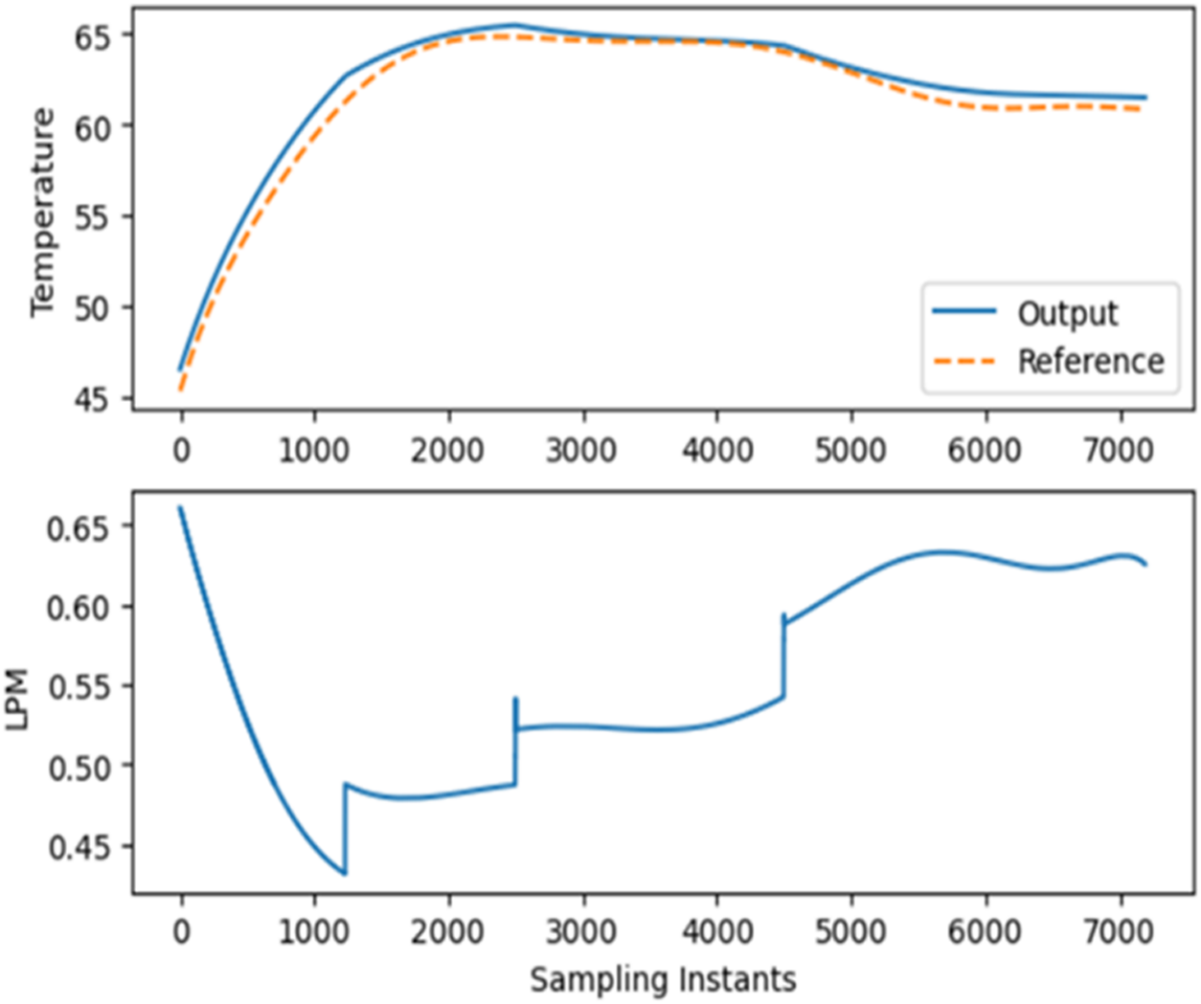
Closed loop trajectory tracking and control signal of RL-NMPC in simulation.

**Table 1:** Comparison of Performance Indices with Class of NMPC

S. No.	MSE	MAE	integral MSE
*P*_NMPC_(*x*)	2.6674	1.5515	2.6674
*P*_SIG_ (*x*)	1.0038	0.9798	1.0038
*P*_RLNMPC_ (*x*)	0.8005	0.6152	0.6103

## 4 Real-Time Validation

The following Figure 16a,b shows the experimental validation of Reinforcement Learning based Nonlinear Model Predictive Controller. The experimental data obtained are filtered to remove the noise signals using smooth data command in MATLAB 2024 version. The simulated Python code was downloaded into the Jetson Orin 8GB board. The processor used is a Jetson Orin Nano SoC with 8GB LPDDR5 RAM with a peak bandwidth of 68 GB/s. It uses an ARM Cortex-A78AE CPU, which contains 6 cores running at 1.5 GHz. It uses the Nvidia Tegra Orin GPU, based on Ampere architecture with 1024 CUDA cores and 32 Tensor cores. It is capable of up to 40 INT8 Sparse TOPs and 20 INT8 Dense TOPs. It has 2 Power Operating modes 7W or 15W. This run was conducted in the 15W mode. Sensors and actuators signals are scaled with factor to covert the signals from 4–20 mA to 0–100% using factors. In this case, the control weights and error weights in NMPC are updated automatically based on the actor and critic feed-forward neural networks. It was observed that the control signal was smooth with the RL-NMPC, further the *T*_*r*_ can be made track closer to the temperature profile by adjusting the number of neurons and layers in the Actor and CRN in near future. It is also observed for one sample of closed loop iteration it takes 165 ms approximately using the Jetson Orin 8GB board.

**Figure 16:**
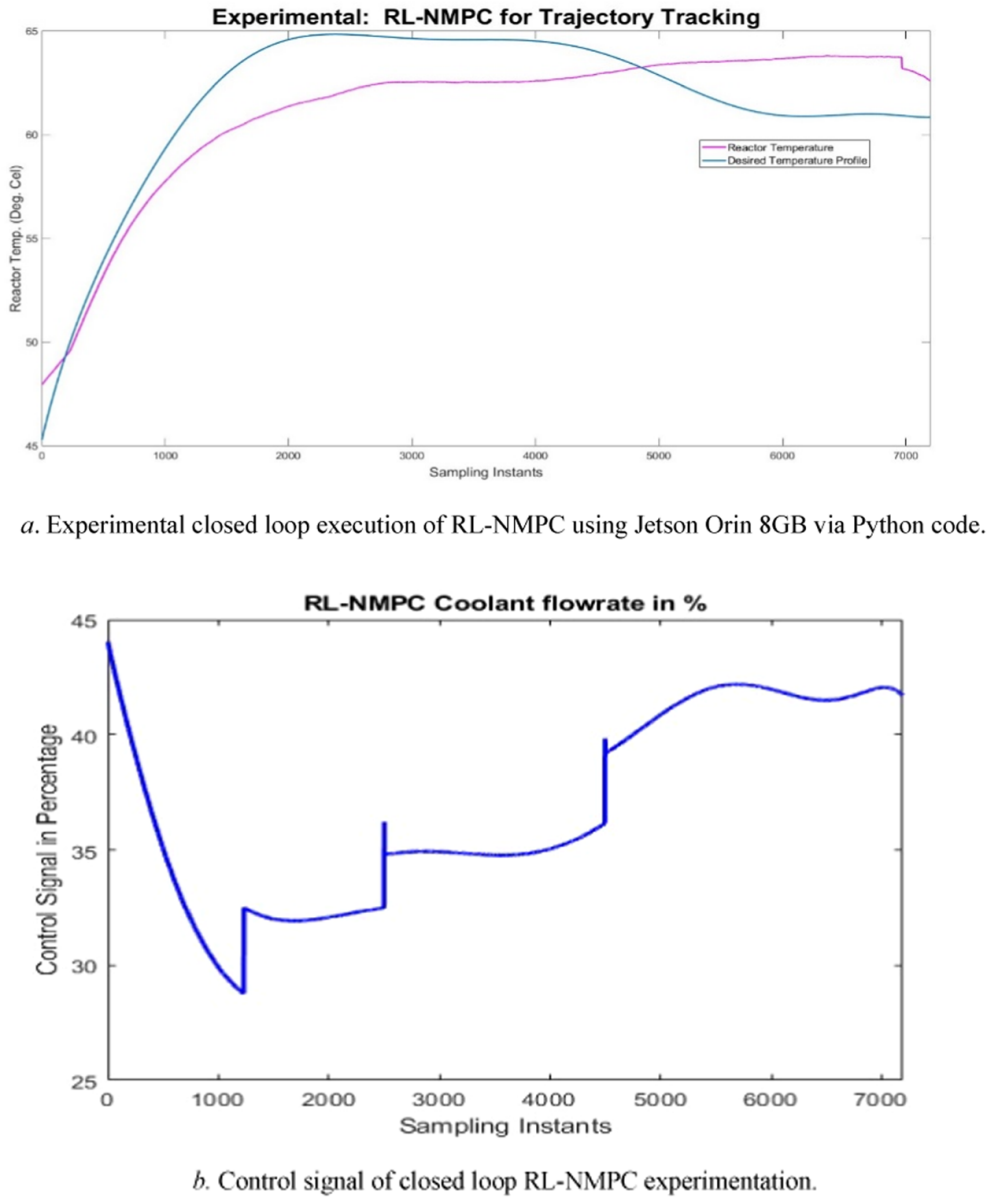
a. Experimental closed loop execution of RL-NMPC using Jetson Orin 8GB via Python code. (b) Control signal of closed loop RL-NMPC experimentation.

Machine learning layers are being integrated into industrial applications, where Jetson technology can be substituted with Digital Twin concepts utilizing cloud-based cyber-physical systems. Gateway-based data acquisition systems can be utilized in real-world plants instead of wireless sensors and actuators, owing to concerns around cyber-attacks.

RL-based NMPC, especially with an Actor-Critic framework, has the potential to improve real-time computational efficiency by shifting optimization to an offline learning phase. However, its deployment on low-power industrial hardware such as PLCs remains a challenge due to memory and processing constraints. Methods such as policy approximation, model compression, and hybrid approaches that combine RL-based learning with explicit control strategies could help bridge the gap between RL-based NMPC and practical industrial implementations.

## 5 Conclusions

The major contribution of this paper is the experimental validation of the RL-based NMPC with dynamic weight updates using the A2C framework via the Jetson Orin 8GB board for trajectory tracking. The tracking performance with RL-NMPC was more stable, showing no undesired fluctuations compared to the results from the RL simulation and experimental tests. Additionally, the Python code in Jetson Orin significantly reduces the computational time for each iteration of RL-NMPC. Jetson Orin implements dynamic weights using an A2C network, a feature that sets it apart from all other NMPC implementations in the literature. Even better performance is expected with sigmoidal weights and its parameter adjustment using A2C.

## Data Availability

The open experimental data used for the modeling of batch reactor are made available under the download section of https://itarasu.com.
